# Methylation of miRNA genes in the response to temperature stress in *Populus simonii*

**DOI:** 10.3389/fpls.2015.00921

**Published:** 2015-10-30

**Authors:** Dong Ci, Yuepeng Song, Min Tian, Deqiang Zhang

**Affiliations:** ^1^National Engineering Laboratory for Tree Breeding, College of Biological Sciences and Technology, Beijing Forestry UniversityBeijing, China; ^2^Key Laboratory of Genetics and Breeding in Forest Trees and Ornamental Plants, College of Biological Sciences and Technology, Beijing Forestry UniversityBeijing, China

**Keywords:** DNA methylation, lipid metabolism, miRNA, *Populus simonii*, temperature stress

## Abstract

DNA methylation and miRNAs provide crucial regulation of the transcriptional and post-transcriptional responses to abiotic stress. In this study, we used methylation-sensitive amplification polymorphisms to identify 1066 sites that were differentially methylated in response to temperature stress in *Populus simonii*. Among these loci, BLAST searches of miRBase identified seven miRNA genes. Expression analysis by quantitative real-time PCR suggested that the methylation pattern of these miRNA genes probably influences their expression. Annotation of these miRNA genes in the sequenced genome of *Populus trichocarpa* found three target genes (*Potri.007G090400*, *Potri.014G042200*, and *Potri.010G176000*) for the miRNAs produced from five genes (*Ptc-MIR396e* and *g*, *Ptc-MIR156i* and *j*, and *Ptc-MIR390c*) respectively. The products of these target genes function in lipid metabolism to deplete lipid peroxide. We also constructed a network based on the interactions between DNA methylation and miRNAs, miRNAs and target genes, and the products of target genes and the metabolic factors that they affect, including H_2_O_2,_ malondialdehyde, catalase (CAT), and superoxide dismutase. Our results suggested that DNA methylation probably regulates the expression of miRNA genes, thus affecting expression of their target genes, likely through the gene-silencing function of miRNAs, to maintain cell survival under abiotic stress conditions.

## Introduction

Temperature has major effects on plant growth and development in the field, where temperatures can change frequently, potentially causing stress on the plant. Heat or cold stress can negatively affect many physiological processes; thus plants have evolved complex signaling pathways that perceive and transduce signals in response to particular stresses (You et al., 2014). These signals act through H_2_O_2_, malondialdehyde (MDA), catalase (CAT), and superoxide dismutase (SOD), which affect the degree of response to abiotic stress (Nie et al., 2015; Zhang et al., 2015). Some of these signals require transcription and are broadly regulated by a variety of factors, including cytosine methylation, covalent modification of DNA by 5-methylcytosines. For example, alterations in methylation might be critical to energy metabolism in the Antarctic polychaete *Spiophanes tcherniai* (Marsh and Pasqualone, 2014). Rakei et al. (2015) identified specific DNA sequences that play an important role in cold tolerance with possible responsive components correlated with cold stress in plants, suggesting that DNA methylation regulates cold stress signals. In addition, the altered methylation state of *CycD3-1* and *Nt-EXPA5* shifted their expression during heat stress in tobacco (Centomani et al., 2015).

MicroRNAs (miRNAs) also regulate expression of genes directly and indirectly related to the response to temperature stress. MiRNAs are small (21–24 nucleotides), non-coding, single-stranded RNAs derived predominantly from intergenic regions, and function as key regulators of gene expression (Sunkar and Zhu, 2004). For instance, work in *Arabidopsis thaliana* identified 29 miRNAs that regulate gene expression in response to drought stress (Hajdarpašić and Ruggenthaler, 2012). Also, in *Larix leptolepis*, four miRNA families (miR159, miRNA169, miRNA171, and miRNA172) are all induced by abiotic stress and their targets regulate genes crucial to cell development, including MYB transcription factors (miR159), an NF-YA transcription factor (miR169), a scarecrow-like transcription factor (miR171) and *apetala2* (miR172; Zhang et al., 2010). In addition, Ishikawa et al. (2014) found that a genotoxic stress-responsive miRNA, miR574-3p, delays cell growth by suppressing the enhancer of a rudimentary homolog gene *in vitro*.

Thus, miRNAs and DNA methylation have crucial functions in regulating gene expression in response to abiotic stress in plants. However, so far, few studies have examined possible mutual adjustments between DNA methylation and miRNAs. Rykov et al. (2013) found that the methylation of *MIR125b-1* and *MIR137* genes was correlated with non-small-cell lung cancer progression. Also, repression of miRNAs was correlated to hypermethylation of their promoters in human cancer cells (Li et al., 2011). Additional research has begun to examine the potential interaction between DNA methylation and miRNAs in response to abiotic stress in plants, but so far this interaction remains unclear.

In comparison to annual plants, perennial plants undergo more temperature changes over their longer lives. Here, we chose *Populus simonii* as an experimental system to examine the possible interaction between cytosine methylation and miRNAs. The advantages of using *Populus* species as genomic models for tree molecular biology have been extensively reported. Among *Populus* species, *P. simonii* shows broad geographic distribution and a strong ability to survive, even in extreme temperatures (-41 to +43°C) and under other abiotic stresses. It is one of the most important native tree species in northern China, for its commercial and ecological value (Wang et al., 2012).

Considering global climate change and frequent extreme weather, including very low and high temperatures, studying the plant response to temperature stress may provide important information for future agricultural and ecological studies. Given the increasing evidence for miRNAs and DNA methylation as important regulators of gene expression in response to abiotic stresses, we hypothesized that the potential interaction of miRNAs and DNA methylation plays a critical role in stress-responsive gene expression. Here, we used *P. simonii*, a resistant and adaptable species, for methylation-sensitive amplification polymorphism (MSAP) and transcript level analysis through quantitative real-time PCR (qRT-PCR) to uncover changes of methylation and expression of miRNA genes in response to heat and cold stress. This study provides new insights on different DNA methylation-mediated regulatory mechanisms in the response to temperature stresses in plants.

## Materials and Methods

### Plant Materials and Treatments

*Populus simonii* ‘QL9’ was planted in pots under natural light conditions (1,250 l mol m^-2^ s^-1^ of photosynthetically active radiation), 25 ± 1°C, 50% ± 1 relative humidity and 12 h day/night in an air-conditioned greenhouse. In this study, thirty annual clones of the same size (50 cm in height) were divided into three groups; one group was chosen to act as the control group and other two groups were treated by heat or cold stress, respectively. These treatment groups were exposed to 42 and 4°C for 3, 6, 12, and 24 h for heat, and cold stress treatments, respectively. The 3- and 6-h time points were chosen to capture early responsive genes, and the 24-h time point for late responsive genes (Lee et al., 2005). Clones not exposed to abiotic stress were used as the control group. Three biological replicates were used for each treatment time point, including the control group. For physiological and gene expression analysis, fresh leaves were collected from the five groups, then immediately frozen in liquid nitrogen and stored at -80°C until analyzed.

### Measurement of Physiological and Biochemical Characteristics

Endogenous H_2_O_2_ levels were detected by measuring luminol-dependent chemiluminescence according to the method described by Dat et al. (1998) and the H_2_O_2_-specific fluorescent probe H2DCF-DA (green; Molecular Probes, Eugene, OR, USA, prepared in a MES-KCl buffer, pH 5.7). The amount of malondialdehyde (MDA), and the activities of SOD and catalase (CAT) were measured by absorption photometry using a spectrophotometer. The details were as described by Giannopolitis and Ries (1977), Carrillo et al. (1992), and Song et al. (2013), respectively.

### DNA and RNA Extraction

Plant materials were stored in liquid nitrogen and total genomic DNA was extracted using a DNeasy Plant Mini kit (Qiagen China, Shanghai), according to the manufacturer’s protocol. Total RNA was extracted using an RNeasy Plant Mini Kit (Qiagen, China, Shanghai) following the manufacturer’s protocol. Genomic DNA and RNA were measured with a Nano Vue UV/visible spectrophotometer (GE Healthcare Company) and stored at -80°C.

### Methylation-sensitive Amplification Polymorphism (MSAP) Analysis

Methylation-sensitive amplification polymorphism analysis was carried out based on an established protocol (Sha et al., 2005; Peredo et al., 2006), and the isoschizomers *Hpa* II and *Msp* I were employed as frequent-cutter enzymes. During the selective PCR step, *Eco*R I and *Hpa* II/*Msp* I primers adding three additional selective nucleotides were used. The selective PCR products were resolved by electrophoresis on 6% sequencing gels and detected with silver staining (Tixier et al., 1997). The differentially amplified fragments represent stress-responsive differentially methylated regions.

The isolation of polymorphic bands was performed as described previously (Sha et al., 2005). Briefly, polymorphic fragments were excised from the gels, hydrated in 50 μl of water, and incubated at 42°C for 30 min. The eluted DNA was amplified with the same primer pairs and under the same conditions used for selective amplification. Sequence information was obtained by cloning the fragments into vector pMD18-T (Takara Bio, Inc., Tokyo, Japan), and three positive clones for each individual were selected for sequencing.

The methylation patterns of specific cytosine loci (5′-CCGG-3′) obtained by MSAP have four types: unknown, unmethylated, methylated at CG (^m^CG) and hemi-methylated at CNG (^m^CNG; **Supplementary Figure S1**). The relative methylation levels were calculated using the following equations: relative methylated level $(\frac{m_{CG}}{unknown+m_{CG}+m_{CNG}+unmethylated}),$ relative hemi-methylated level $(\frac{m_{CNG}}{unknown+m_{CG}+m_{CNG}+unmethylated}),$ and relative total methylation level $\left(\frac{m_{CG}+m_{CNG}}{unknown+m_{CG}+m_{CNG}+unmethylated}\right)$. In these formulas, ^m^CG represents the number of MSAP markers with the ^m^CG methylation pattern. Similarly, ^m^CNG, unknown, and unmethylated represent the number of MSAP markers with ^m^CNG, unknown, and unmethylated methylation patterns, respectively.

### Bisulfite Sequencing of Candidate Differentially Methylated Sequences

For analysis of the candidate differentially methylated sequences (DMSs), genomic DNA was treated with bisulfite and used as template for amplification, which was carried out for 35 cycles. The assay primers span the region that contains the 5′-CCGG-3′ sites and are listed in **Supplementary Table S3**. PCR products were then purified using a Gel Extraction Kit (Qiagen, Hilden, Germany) and cloned into the vector pMD18-T (Takara Bio, Inc., Tokyo, Japan). 20 positive clones for each individual were selected for sequencing through an ABI sequencer (PRISM BigDye Terminator, ABI, Sunnyvale, CA, USA).

### Annotation of miRNA and Target Genes

The DMSs were analyzed using miRBase^1^ to find the mapped miRNA genes and psRNATarget tools^2^ to map the target genes in the sequenced reference genome of *P. trichocarpa*. The annotation information for target genes was obtained from PopGenIE^3^ and pathway analysis of biological process from KEGG^4^.

### Real-time Quantitative PCR of Mature miRNAs

Quantitative PCR analysis of miRNA was carried out following a high-stringency protocol where a poly A tail was added by using poly A polymerase. The Power SYBR Green PCR Master Mix (ABI) and the StepOne+ Real-Time PCR System (ABI) were used to perform quantitative PCR according to the standard protocol. The forward primers were designed based on miRNA sequences in miRBase 21.0^5^. The reverse primer was designed based on the poly(T) adapter, which was always the same (5′-GTCGTATTAATTCTGTGCTCGC-3′). The internal reference gene was a 5.8S rRNA (forward primers: 5′-GTCTGCCTGGGTGTCACGCAA-3′; **Supplementary Table S2**).

### Gene Expression Analysis by qRT-PCR

For quantitative PCR analysis, the TaKaRa ExTaq R PCR Kit, SYBR green dye (TaKaRa, Dalian, China) and a DNA Engine Opticon 2 machine (MJ Research) were used. Gene-specific primers were designed to target the 3′ UTR of each gene (**Supplementary Table S1**). A melting curve was used to check the specificity of each amplified fragment. For all reactions, triplicate technical and biological repetitions of each individual were performed; the PCR was performed according to Song et al. (2014). After amplification, the PCR products were sequenced to check the specificity of the primer sets. Relative expression levels of candidate genes were standardized to the transcript levels for *PsiACTIN*, which shows stable expression under abiotic stress calculated by the 2–^ΔΔ^Ct method.

### Validation by Degradome Sequencing

We used the fastx toolkit to exclude low-quality reads from the degradome sequencing and to remove adapter sequences. The Cleaveland 2.0 software was used to further analyze the reads. Briefly, the *P. trichocarpa* transcripts database of JGI Phytozome V 7.0 was mapped with the reads. Next, the true miRNA cleavage site was distinguished from background noise with a target plot. We used the default parameters and performed 100 randomized sequence shuﬄes with Cleaveland 2.0. The cleaved targets were categorized into five categories using the following criteria: (1) the read abundance of the cleavage site had the maximum value on the transcript; (2) the read abundance of the cleavage site had the maximum value on the transcript but was not unique; (3) the read abundance of the cleavage site did not have the maximum value but was higher than the median on the transcript; (4) the read abundance of the cleavage site was equal to or less than the median on the transcript; and (5) only one raw read existed at the cleavage site. Only the targets that were verified by degradome sequencing were recorded in this study.

### Statistical Analysis

Significance of differences in enzyme activities were determined with the Least Significant Difference test using SPSS 20 (Copyright IBM Corporation 1989). Differences were considered statistically significant when *P* < 0.05. Asterisks “^∗^” represent *P* < 0.05, “^∗∗^” represent *P* < 0.01 compared with control group.

## Results

### Changes of Physiological and Biological Parameters in Response to Temperature Stress

**FIGURE 1:**
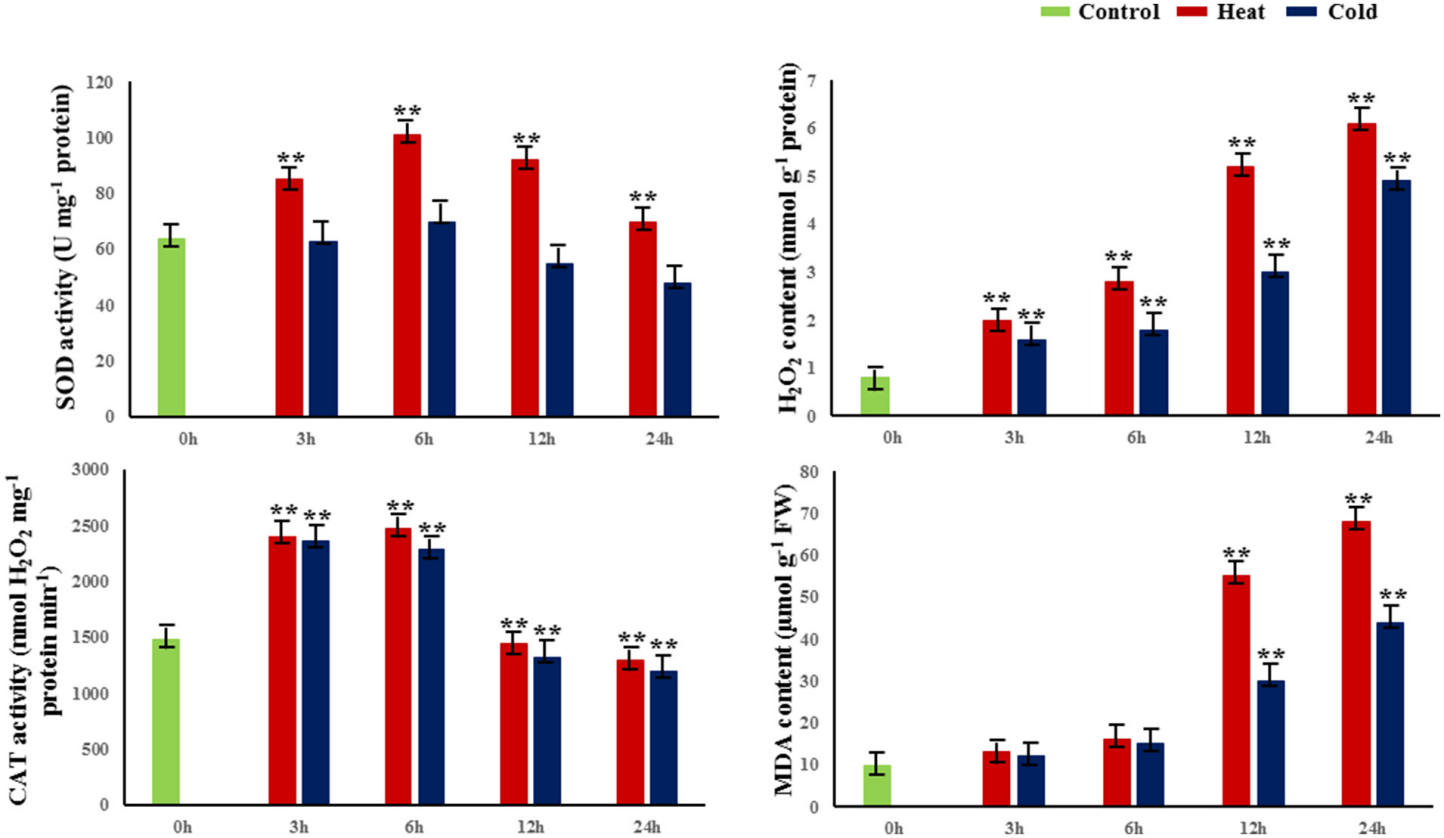
**Changes in physiological and biochemical properties in response to abiotic stress.** SOD superoxide dismutase, H_2_O_2_ hydrogen peroxide, MDA malondialdehyde, CAT catalase. 0 h indicates the control group without stress treatment. 3–24 h indicate different times of exposure to heat and cold stress. Error bars represent standard error. Activities are presented as mean ± SE, and *n* = 3. An asterisk indicates differences at ^∗^*P* < 0.05 compared with the control group, and two asterisks indicates significant differences at ^∗∗^*P* < 0.01 compared with the control group.

To evaluate the dynamic biological and physiological reactions that occur during temperature treatment, we treated 30-cm cuttings from 1-year-old branches at 42 or 4°C for 0, 3, 6, 12, and 24 h and measured four parameters of the cuttings: H_2_O_2_ contents, and activities of catalase (CAT), SOD, and malondialdehyde (MDA; **Figure 1**). In both heat and cold stress treatments, the activity of CAT and SOD reached a peak at 6 h; also, CAT and SOD activities were significantly higher under heat stress than cold at 6 h. In addition, the amounts of MDA and H_2_O_2_ significantly increased from 12 to 24 h in cold- and heat-treated plants (*P* < 0.01; **Figure 1**).

### Relative Levels and Patterns of Cytosine Methylation under Temperature Stress

**FIGURE 2:**
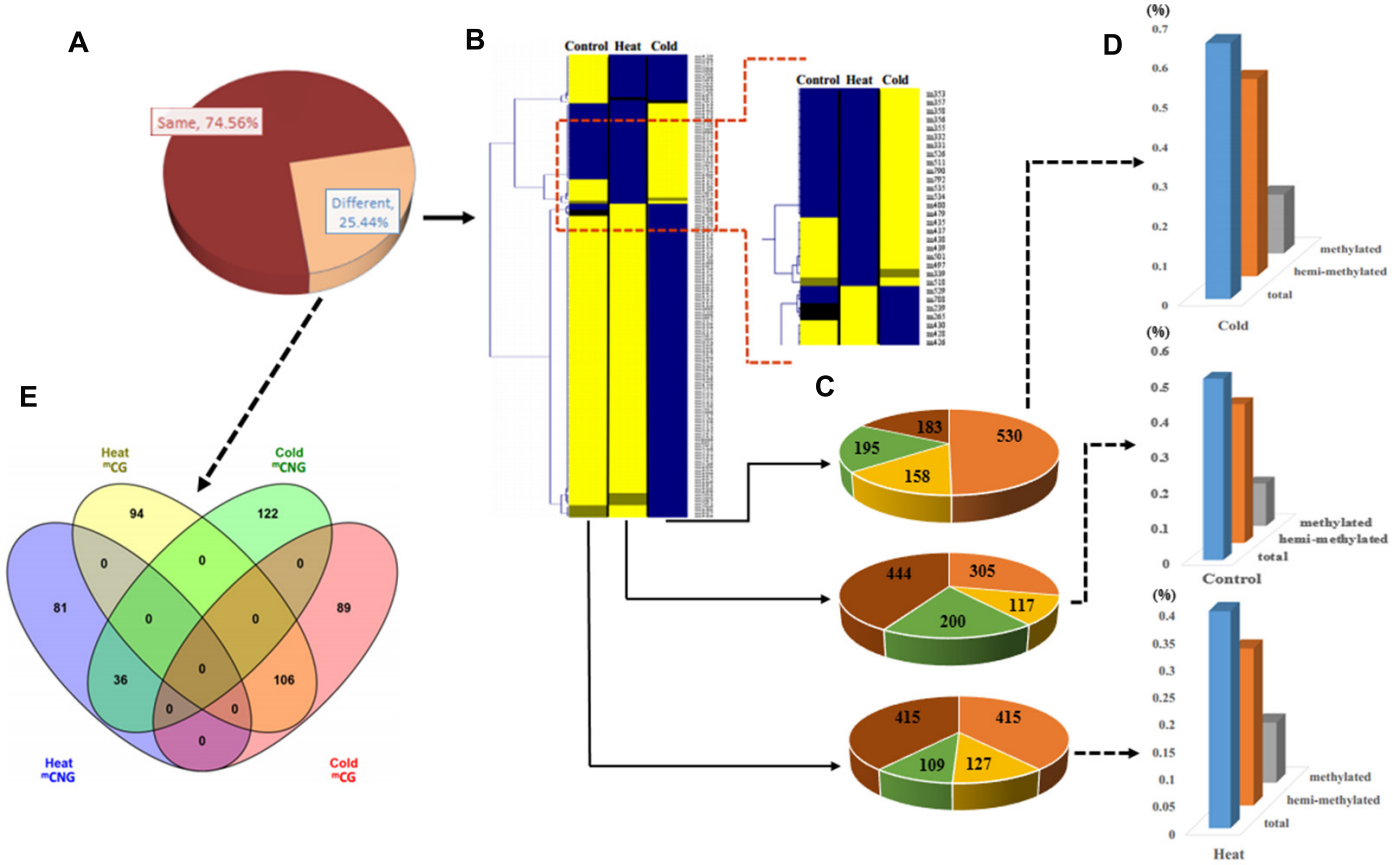
**Analysis of methylation patterns and levels in *Populus simonii* under heat stress, cold stress, and in the control group.** **(A)** The ratio of different MSAP band types: the dark red part represents MSAP bands with the same methylation pattern in heat, cold, and control groups; the orange part represents MSAP bands with different methylation patterns in heat, cold, and control groups. **(B)** Hierarchical clustering analysis of pattern of MSAP markers: 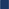 represents unknown methylation pattern; 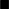 represents hemi-methylated pattern; 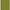 represents methylated pattern; 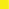 represents unmethylated pattern. **(C)** The number of four MSAP patterns: 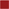 represents unknown methylation pattern; 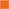 represents hemi-methylated pattern; 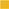 represents methylated pattern; 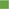 represents unmethylated pattern. **(D)** Relative methylation level, relative hemi-methylated level, and relative total methylation level (see Materials and Methods for calculations). **(E)** Venn diagram showing the number of heat stress and cold stress loci with stress-specific DNA methylation.

We used samples at 6 h of treatment to examine DNA methylation changes and gene expression responses underlying these treatments. MSAP analysis, which can reveal different methylation sites in the genome, was used to examine the genome-wide patterns of DNA methylation in response to temperature stress treatments. We used 240 primer combinations with 15 *Hpa* II/*Msp* I and 20 *Eco*R I primers (Excel S1) to detect the sites of DNA methylation at the 5′-CCGG-3′ sequence from *P. simonii*. In total, MSAP produced 4199 methylation bands, including 1066 polymorphic loci (25.39%; **Figure 2A**). Among these, 70.73 and 46.90% polymorphic loci were from cold stress and heat stress, respectively.

To estimate the relative methylation levels under different abiotic stresses, we next examined the methylation state (unknown, unmethylated, methylated at CG, or hemi-methylated at CNG) of the methylation bands (**Supplementary Figure S1**). Relative total methylation levels ranged from 39.59% (heat) to 64.54% (cold) and the relative total methylation level of the control group was 50.84%. The relative methylation level (^m^CG) was 10.98% in heat-treated plants, 14.82% in cold-treated plants, and 11.91% in control plants. In addition, the relative hemi-methylation level of *P*. *simonii* in different treatments was 28.61% (heat), 49.72% (cold), and 38.93% (control). These results showed that plants showed the highest relative methylation in all methylation patterns under cold stress treatment, and the lowest level under heat stress treatment (**Figures 2B–D**). Comparison of methylation band types under heat and cold stress treatments also detected differences in the cytosine methylation patterns under different abiotic stresses. This identified 175 heat-specific bands (94 ^m^CG and 81 ^m^CNG), 211 cold-specific methylated bands (89 ^m^CG and 122 ^m^CNG), and 142 bands present in both heat and cold (106 ^m^CG and 36 ^m^CNG; **Figure 2E** and Excel S1).

### Identification of miRNA Genes Methylated in Response to Temperature Stress

Based on the polymorphisms observed in MSAP bands under different temperature stresses, we isolated and sequenced a subset of the stress-specific DMSs. We focused on two classes: Class I includes DMSs present in both heat and cold stresses, but not in the control group; Class II includes DMSs specific to heat or cold stress. From the 1066 differentially methylated bands, we isolated and sequenced 400 bands, including 150 stress-specific MSAP bands for each stress and 100 bands common to both stresses. After removing low-quality sequences and redundant fragments, we finally obtained 259 DMSs.

To identify miRNA genes potentially affected by methylation in response to temperature stress, the DMSs were annotated by BLAST searches against miRBase. This identified seven DMSs that mapped to conserved miRNA genes in the sequenced *P. trichocarpa* genome: *Ptc-MIR156i*, *Ptc-MIR156j*, *Ptc-MIR167h*, *Ptc-MIR390c*, *Ptc-MIR393a*, *Ptc-MIR396e*, and *Ptc-MIR396g* (Excel S2); these are homologs of the *P. simonii* miRNA genes. The psRNATarget algorithm predicted 111 targets of the miRNAs produced by these methylated miRNA genes (Excel S3) and gene ontology (GO) classification of these miRNA targets showed enrichment of hydrolase activity, acting on acid anhydrides, ribonucleotide binding, purine nucleotide binding, DNA binding, and purine nucleoside binding in molecular function GO terms (**Supplementary Figure S3A**). In biological process, regulation of cellular biosynthetic process, RNA metabolic process, regulations of macromolecule biosynthetic process, and regulation of gene expression were enriched (**Supplementary Figure S3B**).

### Methylation of miRNA Genes in Response to Temperature Stress

**FIGURE 3:**
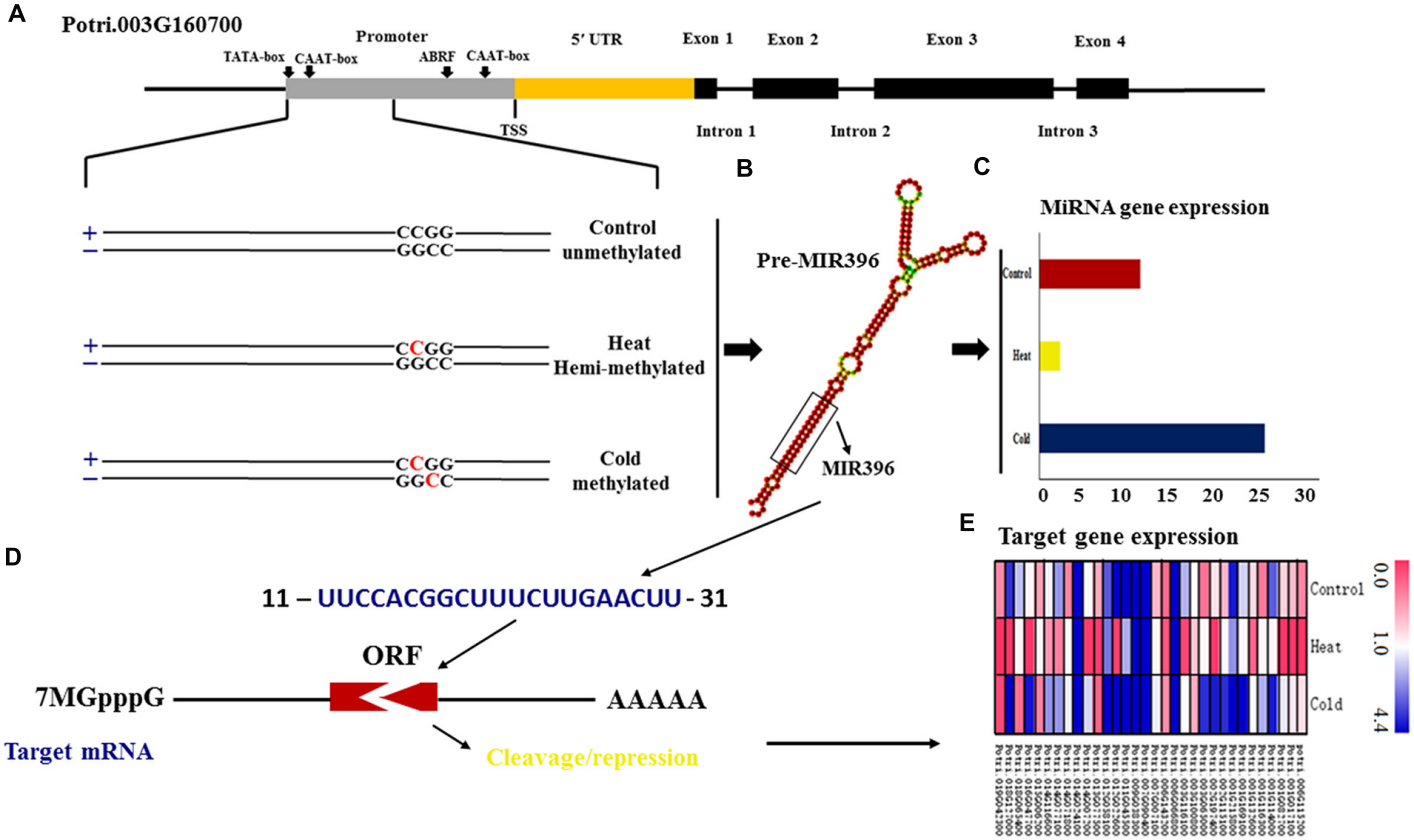
**DNA methylation changes in miRNA genes in *P. simonii* under heat, cold and control conditions.** **(A)** Schematic representation of the differentially methylated patterns of *MIR396g*, whose host gene is *Potri.003G160700*. Horizontal lines represent the gene sequence. The red C represents 5-methylcytosine and black C represents normal cytosine; **(B)** The secondary structure of the miRNA396g precursor. **(C)** The expression of the methylated *Psi*-*MIR396g* locus. **(D)** The interaction between miR396g and its target gene. **(E)** The expression of target genes regulated by miR396g in response to heat and cold, and in the control group.

Annotation analysis of all target genes based on their homologs in the sequenced reference genome of *P. trichocarpa* showed that of the three miRNA genes are located in genic regions. Examination of the local methylation patterns of these miRNA genes revealed that *Ptc-MIR393a* shows different cytosine methylation patterns among control group (^m^CNG), cold (^m^CG), and heat stress (^m^CNG) and is located in the 5′ untranslated regions (UTR) and first exon of *Potri.008G062800*, which has two exons and one intron. Also, *Ptc-MIR396e* is located in the 5′ UTR and first exon of *Potri.018G127000*, which has two exons and one intron and was unmethylated in the control group, ^m^CG in cold-treated plants, and ^m^CNG in heat-treated plants. Moreover, *Ptc-MIR396g* is located in the promoter of *Potri.003G160700*, which contains four exons and three introns, and was unmethylated in the control group, ^m^CG in cold- and ^m^CNG in heat-treated plants (**Figure 3A** and **Table 1**).

**Table 1:** Methylation pattern and expression of miRNA genes.

miRNA ID	Methylation pattern	Expression of miRNA genes (Fold change)
	Control group	Cold	Heat	Control group	Cold	Heat
MIR156i	Unmethylated	CG	CNG	102.90 , 4.12	36.92 , 1.18	142.74 , 5.00
MIR156j	Unmethylated	CNG	CNG	102.70 , 3.29	36.88 , 0.89	142.81 , 5.14
MIR167h	Unmethylated	CG	CNG	3.15 , 0.07	1.11 , 0.05	11.61 , 0.24
MIR390c	Unmethylated	Unmethylated	CG	0.39 , 0.00	10.67 , 0.39	0.81 , 0.03
MIR393a	CNG	CG	CNG	22.87 , 1.07	13.48 , 0.67	24.15 , 1.11
MIR396e-3p	Unmethylated	CG	CNG	9.46 , 0.24	7.96 , 0.27	19.63 , 0.24
MIR396e-5p	Unmethylated	CG	CNG	871.26 , 28.75	576.08 , 19.01	1623.14 , 32.46
MIR396g-3p	Unmethylated	CG	CNG	2.76 , 0.04	2.15 , 0.06	4.33 , 0.04
MIR396g-5p	Unmethylated	CG	CNG	228.27 , 4.11	117.05 , 5.17	250.22 , 8.25


The cytosine methylation sites cut by *Eco*R I and *Hpa* II/*Msp* I also are located in intergenic regions in *Ptc-MIR156i*, *Ptc-MIR156j*, *Ptc-MIR167h*, and *Ptc-MIR390c*. Therefore, we used these sites to assay methylation in these regions. The *Ptc-MIR156i* and *Ptc-MIR156j* loci were unmethylated in the control group, while *Ptc-MIR156i* had ^m^CG in cold-treated plants, and ^m^CNG in heat-treated plants, but *Ptc-MIR156j* had ^m^CNG in cold- and heat-treated plants. In addition, *Ptc-MIR167h* was unmethylated in the control group, had ^m^CG in cold-treated plants, and had ^m^CNG in heat-treated plants. Also, *Ptc-MIR390c* was unmethylated in the control group and cold-treated plants, but had ^m^CG in heat-treated plants (**Table 1**). Thus, these miRNA genes showed different patterns of methylation, but most of the miRNA genes (except for *Ptc-MIR156j* and *Ptc-MIR390c*) were methylated at CG sites under cold stress. By contrast, under heat stress, these miRNA genes (except for *Ptc-MIR390c*) were methylated at CNG sites.

To confirm the status of the methylation of DMSs, we performed bisulfite sequencing for each of the temperature stresses (treated for 6 h) and the control group. The methylation level of CHG on candidate DMSs ranged from 34.2 to 100.0% in the control, CHH ranged from 48.6 to 69.5%, and CG ranged from 51.3 to 90.6%. The methylation levels in the temperature treated samples are listed in **Table 2**, and the sequences contained less CG and CHG methylation than CHH methylation. In treated individuals, *de novo* methylation and demethylation occurred simultaneously (Excel S4 and **Supplementary Figure S5**), but methylation of these candidate sequences showed a decreasing trend, with the methylation under heat treatment declining more than under cold treatment, and the methylation level in ^m^CNG context being lower than in ^m^CG and unmethylated.

**Table 2:** Methylation level in different sequence contexts.

Sequence	Sample	Methylation pattern^b^	^m^CG/total CG	Number of CG	^m^CHG/total CHG	Number of CHG	^m^CHH/total CHH	Number of CHH
1^a^	Control	Unmethylated	105/140 (75.0)^c^	7	123/140 (87.9)	7	623/900 (69.2)	45
	Heat-treated	^m^CNG	99/140 (70.7)	7	81/140 (57.9)	7	400/900 (44.4)	45
	Cold-treated	^m^CG	63/140 (45.0)	7	120/140 (85.7)	7	601/900 (66.8)	45
2^a^	Control	Unmethylated	145/160 (90.6)	8	64/120 (53.3)	6	308/560 (55.0)	28
	Heat-treated	mCNG	101/160 (63.1)	8	66/120 (55.0)	6	199/560 (35.5)	28
	Cold-treated	mCNG	116/160 (72.5)	8	78/120 (65.0)	6	411/560 (73.4)	28
3^a^	Control	Unmethylated	144/200 (72.0)	10	57/80 (71.3)	4	261/420 (62.1)	21
	Heat-treated	^m^CNG	145/200 (72.5)	10	18/80 (22.5)	4	162/420 (38.6)	21
	Cold-treated	^m^CG	116/200 (58.0)	10	55/80 (68.8)	4	288/420 (68.6)	21
4^a^	Control	Unmethylated	226/260 (86.9)	13	100/100 (100.0)	5	482/760 (63.4)	38
	Heat-treated	^m^CG	167/260 (64.2)	13	38/100(38.0)	5	383/760 (50.4)	38
	Cold-treated	Unmethylated	199/260 (76.5)	13	84/100 (84.0)	5	517/760 (68.0)	38
5^a^	Control	^m^CNG	82/160 (51.3)	8	123/140 (87.9)	7	343/580 (59.1)	29
	Heat-treated	^m^CNG	80/160 (50.0)	8	120/140 (85.7)	7	144/580 (24.8)	29
	Cold-treated	^m^CG	118/160 (73.8)	8	99/140 (70.7)	7	247/580 (42.6)	29
6^a^	Control	Unmethylated	222/260 (85.4)	13	41/120 (34.2)	6	243/500 (48.6)	25
	Heat-treated	^m^CNG	149/260 (57.3)	13	35/120 (29.2)	6	138/500 (27.6)	25
	Cold-treated	^m^CG	156/260 (60.0)	13	15/120 (12.5)	6	269/500 (53.8)	25
7^a^	Control	Unmethylated	120/220 (54.5)	11	62/120 (51.7)	6	445/640 (69.5)	32
	Heat-treated	^m^CNG	63/220 (28.6)	11	41/120 (34.2)	6	280/640 (43.8)	32
	Cold-treated	^m^CG	105/220 (47.7)	11	63/120 (52.5)	6	361/640 (56.4)	32


*^a^The number represents candidate differentially methylated sequences (DMSs), seen in Excel S4. ^b^The methylation pattern refers to the pattern derived from DMSs cut by *Eco*R I and *Hpa* II/*Msp* I. ^c^The sum of m^*5*^C in the total methylatable Cs in the indicated sequences in a total clones examined are presented as numbers observed and as percentages (%) in parentheses. For example, the 140 CG sites were calculated by multiplying 20 clones by seven sites derived from candidate DMS 1 overlapping with *Ptc-MIR156i* (Excel S2).*

### Expression of miRNA Genes in Response to Temperature Stress

To verify the possible relationship between DNA methylation pattern and expression of candidate miRNA genes, we used quantitative real-time PCR (qRT-PCR) to detect the expression levels of these miRNA genes in control and treated samples (**Supplementary Table S2**). Five miRNA genes (*Ptc-MIR156i, Ptc-MIR167h, Ptc-MIR393a, Ptc-MIR396e*, and *Ptc-MIR396g*) showed ^m^CG modification under cold stress and ^m^CNG modification under heat stress but were unmethylated in the control group (**Table 1**). The qRT-PCR analysis showed that the expression of miRNA genes with ^m^CNG was significantly higher than the miRNA genes with ^m^CG and also higher than the non-methylated genes under temperature stress. *Ptc-MIR390c* showed ^m^CG under heat stress and was unmethylated in cold-treated and control groups and also showed lower expression when modified with ^m^CG, compared with the unmethylated control (**Table 1**; **Figures 3B,C**). Thus, generally, under temperature stress, the candidate methylated miRNA genes that had ^m^CNG showed higher expression than those that had ^m^CG. However, miRNA genes with the same methylation pattern under heat and cold stresses showed different expression levels under these two conditions. For instance, *MIR156j* had the same methylation pattern (^m^CG) in both heat and cold stresses, but it showed higher expression under heat stress than under cold stress (**Table 1**).

### Expression of Target Genes of miRNAs

After we measured the expression of the miRNA genes (**Supplementary Table S1**), we next measured the expression of their target genes to examine the regulatory relationship between the miRNAs and their targets. In total, we found 111 target genes, including 38 targets of Ptc-miR156i/j, 30 of Ptc-miR167h, 11 of Ptc-miR393a, and 32 of Ptc-miR396e/g. To detect whether the changes in miRNA gene expression affected target gene expression, we used qRT-PCR to measure the mRNA levels of the targets of these miRNA genes (**Figure 3E** and Excel S3). The results showed that the targets’ expression varied with the expression changes in the corresponding miRNA genes in response to heat and cold stresses. For example, *Potri.001G294400* encoding a homolog of *Arabidopsis* VACUOLAR SORTING RECEPTOR 5 (VSR5), a target of Ptc-miR156i and j with ^m^CG methylation modification, was downregulated in response to cold stress, when *Ptc-MIR156i* and *j* were upregulated. *Potri.001G323100* encodes a homolog of *Arabidopsis* AUXIN SIGNALING F-BOX 2 (AFB2), is a target of Ptc-miR393a, showed ^m^CNG, and was repressed under heat stress, in contrast to *Ptc-MIR393a*. In short, expression of the target genes decreased as the expression of the corresponding miRNA increased, consistent with the gene-silencing function of miRNAs (**Figure 3D**).

### Regulatory Network of DNA Methylation in the Response to Temperature Stress

**FIGURE 4:**
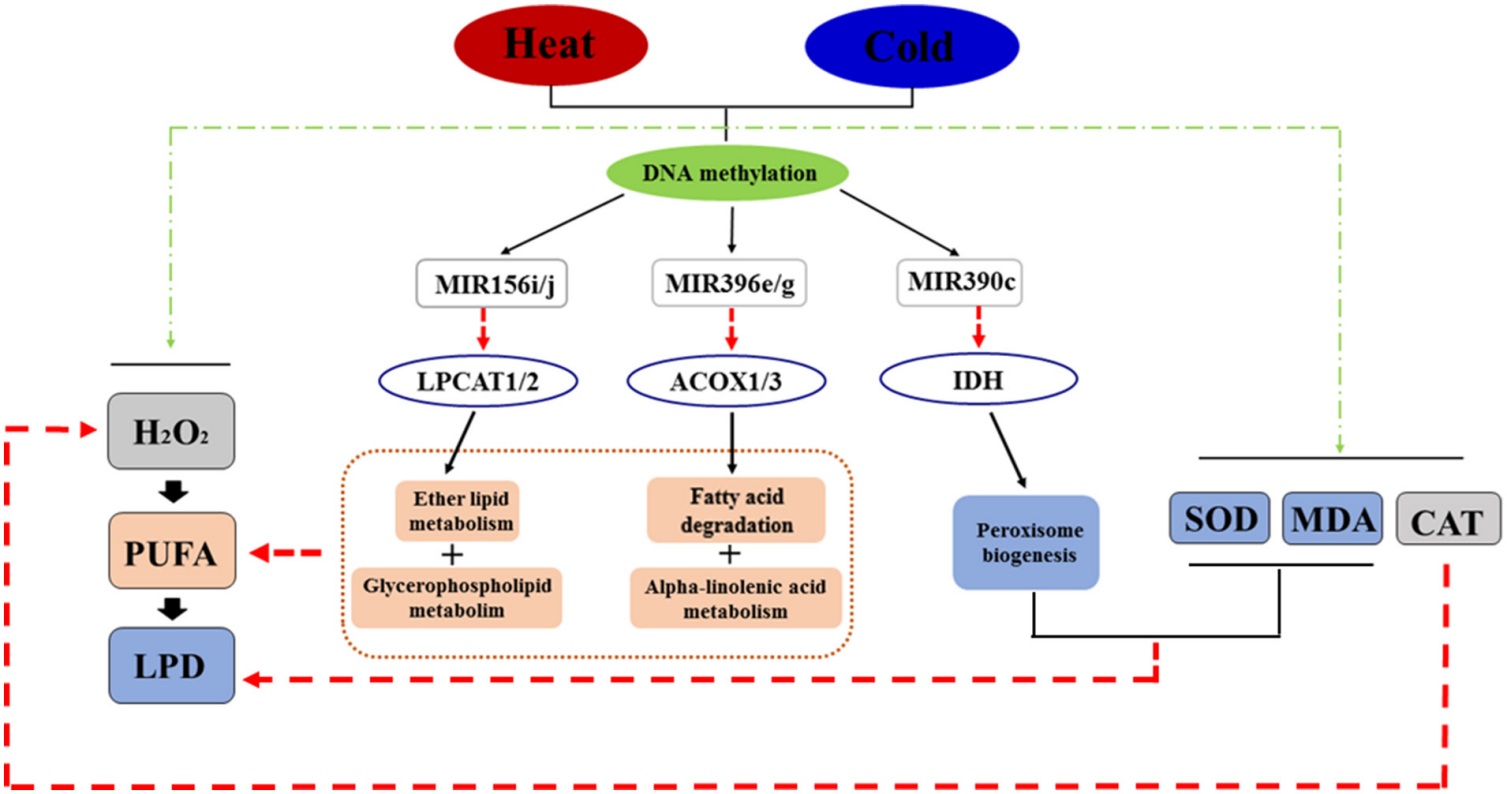
**The regulatory network in response to temperature stress in *P. simonii*.** Black line arrows represent regulatory effects (positive or negative regulation), red line arrows represent negative regulation, and green line arrows represent positive regulation.

KEGG pathway analysis was used to characterize the functions of the target genes and indicated that target genes generally participate in lipid metabolism, including fatty acid degradation. For example, *Potri.007G090400*, a target of Ptc-miR396e and g, encodes a protein that functions in glycerophospholipid metabolism. *Potri.014G042200*, a target gene of Ptc-miR156i and j, encodes a protein involved in ether lipid metabolism. Other predicted products function in alpha-linolenic acid metabolism [*ACYL-COA OXIDASE (ACOX1* and *ACOX3)*] and biosynthesis of unsaturated fatty acids [*ACYL-COA OXIDASE (ACOX1* and *ACOX3)*], which all deplete lipid peroxide. Also, *ISOCITRATE DEHYDROGENASE (IDH)* participates in peroxisome biogenesis (a target gene of Ptc-miR390c; Biswas and Mano, 2015; **Figure 4** and Excel S3). To validate the predicted target genes of miRNAs, degradome sequencing was performed, which confirmed *LPCAT1/2, ACOX1/3*, and *IDH* as targets of *MIR156i/j*, *MIR396e/g*, and *MIR390c*, respectively, in lipid metabolism or peroxidation (**Supplementary Figure S4**).

Based on these pathways and the interactions between DNA methylation, miRNAs, and target genes, we constructed a regulatory network showing the changes of DNA methylation in response to temperature stress (**Figure 4**). In this network, temperature stress (heat and cold) induced production of H_2_O_2_, which produced more oxyradicals attacking polyunsaturated fatty acids, which induced lipid peroxidation to destroy cells. Moreover, heat and cold stresses induced SOD, MDA, and CAT activities to decrease superoxide, lipid peroxide, and H_2_O_2_ levels, respectively. Temperature stress also changed the methylation patterns of specific loci associated with miRNA genes responding to stress treatments, including *Ptc-MIR156i* and *j, Ptc-MIR390c*, and *Ptc-MIR396e* and *g*, which decrease the products of *ACYL-COA OXIDASE (ACOX1* and *ACOX3)*, *PHOSPHOLIPID/GLYCEROL ACYLTRANSRASE FAMILY PROTEIN (LPCAT1* and *LPCAT2)*, and *ISOCITRATE DEHYDROGENASE (IDH)* from *Potri.007G090400*, *Potri. 014G042200*, and *Potri.010G176000*, respectively (Excel S3). These products participate in lipid metabolism and peroxisome biogenesis; these reversible pathways can consume polyunsaturated fatty acids and lipid peroxides to prevent cell death (**Figure 4**).

### Verification of MSAP Data

To make sure the MSAP data are reliable, we used methyl-sensitive PCR (MS-PCR) to confirm the data produced by MSAP. For this purpose, we design MS-PCR primers for 45 MSAP sequences for 15 stress-specific MSAP bands and 15 common MSAP bands in both heat and cold stress treatments (**Supplementary Figure S2**). The MS-PCR results indicate that MSAP is a stable, effective, and reproducible technology for detecting methylated sites that change in response to temperature stress in the genome of *P. simonii*.

## Discussion

Catalase, SOD, and MDA are three important factors participating in general physiological and biochemical processes in plants. H_2_O_2_ produces oxyradicals that attack polyunsaturated fatty acids, thus inducing lipid peroxidation to destroy cells (Roach et al., 2015). CAT metabolizes H_2_O_2_ to prevent or reduce these harmful effects (Nie et al., 2015; Zhang et al., 2015), and provides a parameter allowing evaluation of the degree of physiological and biochemical effects of stress. The decomposition products of lipid peroxide induce cell damage and can be measured by the amount of MDA, which reflects the extent of lipid peroxidation and thus the extent of cell damage. Measurements of MDA and SOD complement each other, as the SOD activity indirectly reflects the oxygen free radical-scavenging ability of the cell, and the level of MDA indirectly reflects the severity of the effects of free radicals on the cell. In this study, we detected changes in CAT activity, SOD activity, MDA contents, and H_2_O_2_ contents in response to temperature stress. These results showed that the physiological reaction of poplar is the strongest after 6-h temperature stress treatment. So, we chose *P. simonii* after 6-h stress treatment as the experimental material for analysis of DNA methylation.

### The Variation of DNA Methylation in Response to Heat and Cold Stress

In this study, we found that 25.38% of methylation sites changed in response to abiotic stress. This was consistent with the observations of Alonso et al. (2015), who examined 49 studies on different abiotic stresses in 18 species; in 78% of studies, the results agreed with the hypothesis that stress elicited changes in global DNA methylation. This suggests that DNA methylation might function in genomic regulation in response to abiotic stress. In addition, Tang et al. (2014) found that cytosine methylation at various loci decreased 10.28% due to drought exposure in *Lolium perenne*. Our results showed that 70.73 and 46.90% of differentially methylated loci responded to cold or heat stress, respectively, suggesting that methylated loci might respond differently to different abiotic stresses. The ^m^CHG and ^m^CHH modifications also showed site-specific methylation between male and female flowers, suggesting that different DNA methylation patterns might have different influences on flower development (Song et al., 2014). By contrast, in this study, we obtained 175 methylated bands (94 ^m^CG and 81 ^m^CNG) that were specific to heat treatment, and 211 methylated bands (89 ^m^CG and 122 ^m^CNG) that were specific to cold stress (**Figure 2E** and Excel S1), suggesting that methylation patterns might differ in the responses to heat and cold stress.

### The Effect of Methylated miRNA Genes on the Expression of Target Genes

Different DNA methylation patterns have different effects on gene expression (Sunkar and Zhu, 2004). *MIR164a* associated with flower development showed significantly lower methylation levels in female flowers than in male flowers and induced expression in male flowers; by contrast, *MIR164a* was only methylated in male flowers and its CHH methylation level was higher than its CG and CHG levels (Song et al., 2014). These results indicated that the expression of miRNA genes might be regulated by their methylation level or pattern. Here, five candidate miRNA genes (*MIR156i, MIR167h, MIR393a, MIR396e*, and *MIR396g*) related to the response to temperature stress showed ^m^CG modification and repressed expression under cold stress; these loci also showed ^m^CNG modification and induced expression under heat stress, compared with the expression of miRNA genes that were unmethylated under control conditions (**Table 1**). This furthermore indicated that the different cytosine methylation patterns of MSAP markers probably associate with different expression levels of miRNA genes.

Moreover, the methylation levels under heat treatment declined more than under cold treatment, which might explain the observation that miRNA expression increased most in heat-treated samples. The CNG methylation was generally lower than the other methylation patterns (^m^CG and unmethylated), which indicated that the results of methylation sequencing support the results of the enzyme digestion. These findings suggest that in poplar, methylation regulates miRNA gene expression in the response to abiotic stress.

In addition, to detect the influence of these miRNA genes on their target genes, we used qRT-PCR to survey the transcript levels of target genes. The results showed that *ACYL-COA OXIDASE 1 (ACOX1)* and *ACYL-COA OXIDASE 3 (ACOX3)*, targets of Ptc-miR396e and g, were induced in cold stress. The *IDH* gene, a target of Ptc-miR390c, was highly expressed in cold stress. Two targets of Ptc-miR156i and j, *PHOSPHOLIPID/GLYCEROL ACYLTRANSFERASE FAMILY PROTEIN (LPCAT1* and *LPCAT2)*, were repressed in heat stress (Excel S3). Degradome sequencing confirmed that these targets produce cleavage products consistent with regulation by the corresponding miRNAs. These variations of expression were consistent with the gene-silencing function of miRNAs, where miRNA levels negatively correlate with the transcript levels of their target genes. Our results suggested that the function of DNA methylation in response to temperature stress might be implemented by affecting expression of miRNAs and their targets.

### The Regulatory Network of *P. simonii* in the Response to Abiotic Stress

Recent work showed that lipid peroxide-derived toxic carbonyl compounds mediate environment stress-induced damage of plants (Biswas and Mano, 2015), suggesting that lipid peroxide might also negatively affect plants in response to abiotic stress. Lipid peroxide derives from lipid peroxidation due to oxyradicals that attack polyunsaturated fatty acids when plants suffer temperature stress that induces oxidative stress. Abiotic stress signals result directly or indirectly from gene expression regulated by many factors, including DNA methylation (Rakei et al., 2015).

Here, we focused on the effect of DNA methylation on regulation of miRNAs as critical to changes in target gene expression to adapt to abiotic stress. Numerous genes related to oxidative stress play crucial roles in maintaining reactive oxygen species homeostasis and levels in organisms. In this study, we found *Ptc-MIR156i* and *j*, and *Ptc-MIR396e* and *g* affected genes related to lipid metabolism and depletion of polyunsaturated fatty acids preventing the production of lipid peroxide. However, miRNAs with different methylation patterns were differentially expressed under different stress treatments, which led to higher expression of *ACOX1* and *3*, and *LPCAT1* and *2* under cold stress with ^m^CG pattern than under heat stress with ^m^CNG pattern. This pattern was reversed for *IDH*, which functions in peroxisome biogenesis directly reducing lipid peroxides. The *ACOX* gene encodes a peroxisomal acyl-CoA oxidase that is thought to catalyze the first reaction during biosynthesis of the fatty acid component of daumones (Joo et al., 2010). The transcript levels of *ACOX* did not show significant changes following oxidative stress induced by perfluorododecanoic acid (Liu et al., 2008). IDH is resistant to denaturation, which reduces its catalytic efficiency in high temperature regimes (Bergmann and Gregorius, 1993). Rzezniczak and Merritt (2012) found that *IDH* seem to be induced by oxidative stress in the enzyme network responsible for the reduction of nicotinamide adenine dinucleotide phosphate and further mentioned that biological network interactions were strongly influenced by environmental conditions. In our study, transcript levels of *ACOX* decreased 2.4-fold and *IDH* decreased over 5-fold in response to heat treatment (Excel S3); these changes of expression might relate to the variation in DNA methylation. Plant *LPCAT* enzymes are crucial in regulating the acyl-CoA composition of cells by transferring polyunsaturated and hydroxy fatty acids produced on phosphatidylcholine straight to the acyl-CoA pool for further metabolism or catabolism (Lager et al., 2013). *Arabidopsis LPCATs* were measured in the reverse reaction from which phosphatidylcholine was transferred to acyl-CoA to a similar extent. Here, *LPCAT1/2* decreased 2.66-fold under heat treatment (Excel S3), while the methylation level of candidate sequences overlapping *Ptc-MIR156i* and *Ptc-MIR156j* declined (**Table 2**), suggesting that the variation of DNA methylation might suppress *LPCAT* correlated with reduced PC for fatty acid desaturation to protect cells from temperature stress. These results indicated that methylated miRNAs might play a key role in *P. simonii* under temperature stress. Our data also provide a series of candidate miRNA genes for research into epigenetic regulation of abiotic stress responses.

## Author Contributions

DZ conceived and designed the experiment. DC performed the DNA and RNA extractions and performed MSAP analyses and drafted the manuscript. YS carried out the gene expression analysis. MT participated in the statistical analyses. All authors read and approved the final manuscript.

## Conflict of Interest Statement

The authors declare that the research was conducted in the absence of any commercial or financial relationships that could be construed as a potential conflict of interest.

## Footnotes

1 http://www.mirbase.org/search.shtml

2 http://plantgrn.noble.org/psRNATarget

3 http://popgenie.org

4 http://www.kegg.jp/kegg/tool/map_pathway2.html

5 http://www.mirbase.org/
