# Supplementary material for: Methylation of miRNA genes in the response to temperature stress in Populus simonii
Source: Front Plant Sci. 2015 Oct 30;6:921. doi: 10.3389/fpls.2015.00921 (PMC4626561; doi:10.3389/fpls.2015.00921)
Supplement: Supplementary file 1 [file Data_Sheet_1.DOC]

***Supplementary Material***

**Methylated miRNA in response to temperature stress in *populous simonii***

**Dong Ci^1, 2^, Yuepeng Song^1, 2^, Min Tian^1, 2^ and Deqiang Zhang^1, 2^***

^1^National Engineering Laboratory for Tree Breeding, College of Biological Sciences and Technology, Beijing Forestry University, No. 35, Qinghua East Road, Beijing 100083, P. R. China

^2^Key Laboratory of Genetics and Breeding in Forest Trees and Ornamental Plants, College of Biological Sciences and Technology, Beijing Forestry University, No. 35, Qinghua East Road, Beijing 100083, P. R. China

*** Correspondence:** Deqiang Zhang, Phone: +86-10-62336007; Fax: +86-10-62336164 Email: [DeqiangZhang@bjfu.edu.cn](mailto:DeqiangZhang@bjfu.edu.cn)

**Supplementary Figures and Tables**

**Figure S1.** Molecular basis of MSAP. Isoschizomers *Hpa* II */Msp* I recognize the same restriction 5’-CCGG site but have different sensitivety to methylation of the cytosines. *Hpa* II digests if the external cytosine is hemimethylated (single-strand) or non-methyled 5’-CCGG site but does not digest if either of the cytosines is fully (double-strand) methylated, while *Msp* I does not, cutting only if the internal cytosine is fully (double-strand) methylated or specifically non-methyled 5’-CCGG site. Neither *Hpa* II nor *Msp* I digests methylation that happened on four cytosines of a 5’-CCGG site (doublestrand) at the same time or some other combinations. Blue represent the methylated cytosines. Green line represents methylation sensitive restriction enzyme digestion. ‘1’and ‘0’ represent statistics of electrophoretic band.


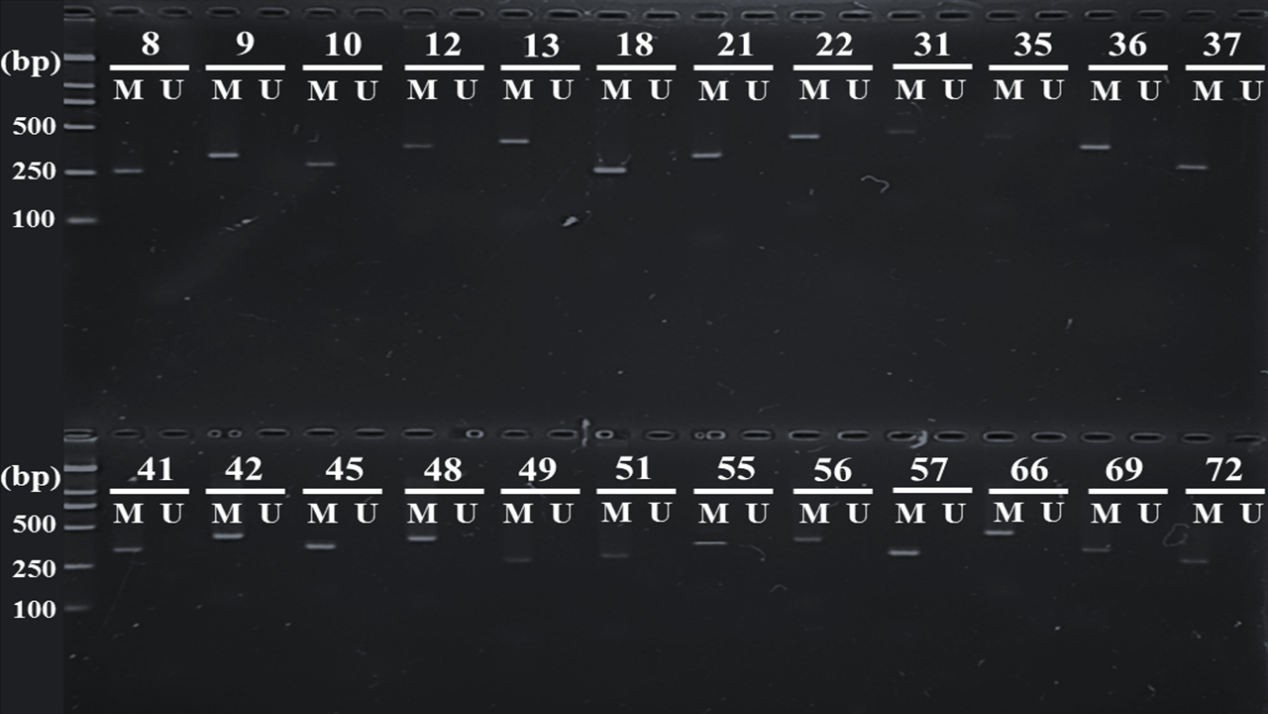


**Figure S2.** Verification of methylation sites using MS-PCR. *8-72* represent methylation fragments MF8-72 (refer to the fragment number in Table S2). *M* methylation primer, *U* unmethylation primer


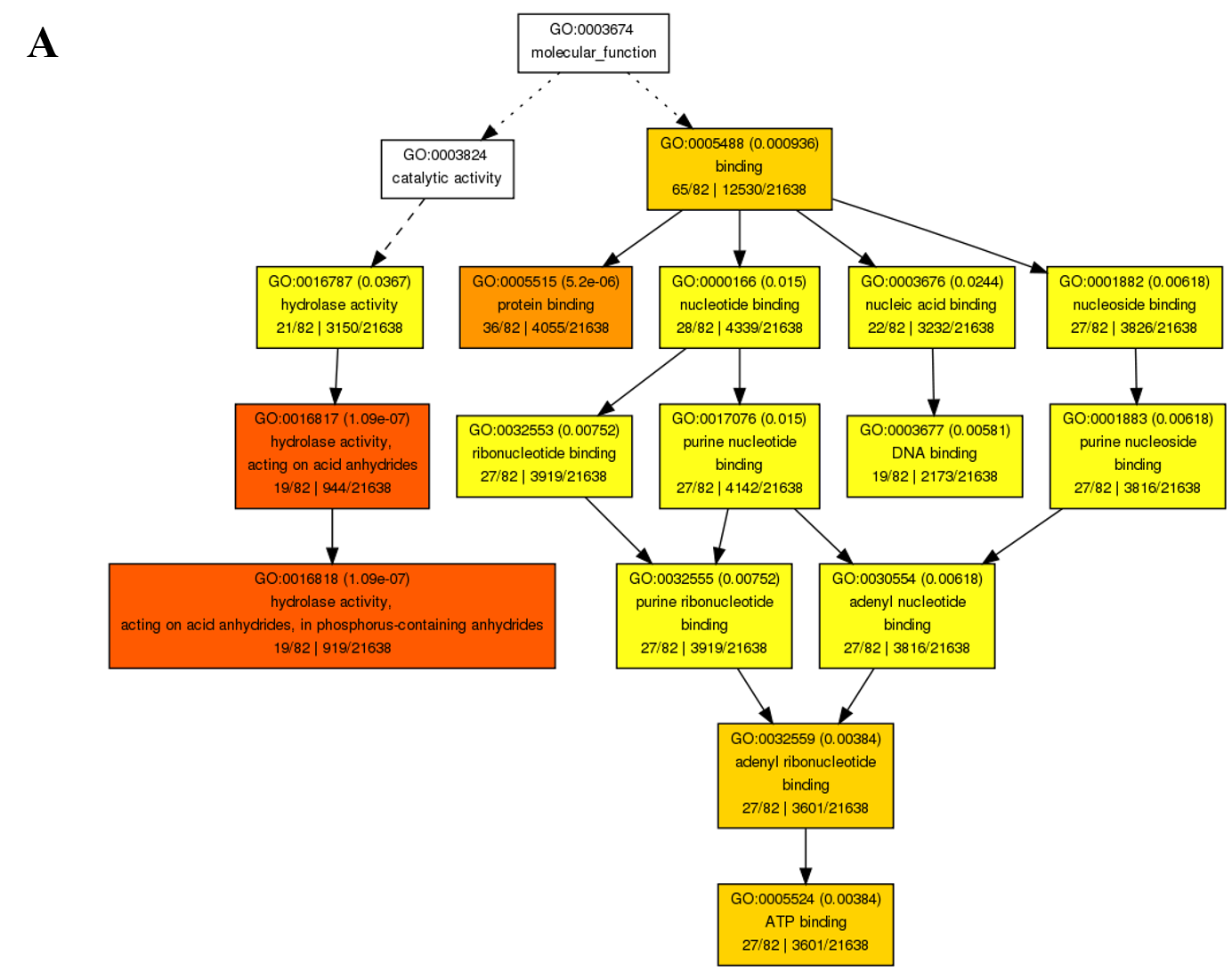


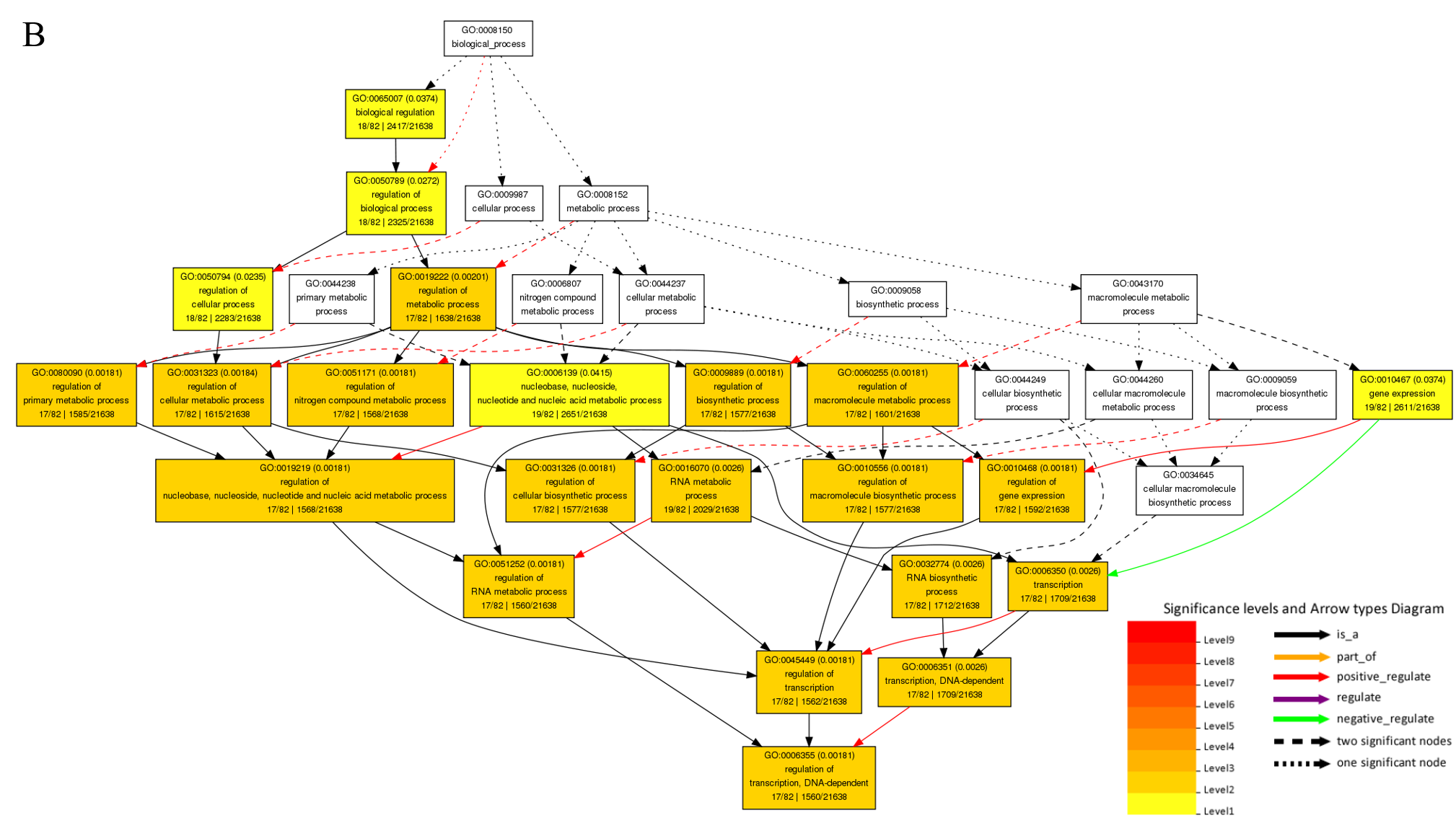


**Figure S3.** Target genes response to temperature stress for statistically enriched GO terms in the ‘Mollecular function’ and ‘Biological process’ ontology. *P*-values <0.05 are reported in parentheses. Colouring of GO term nodes is proportional to their significance as indicated by the scale. A represents AgriGO molecular function analysis of target genes under temperature stress. B represents AgriGO biological process analysis of target genes under temperature stress


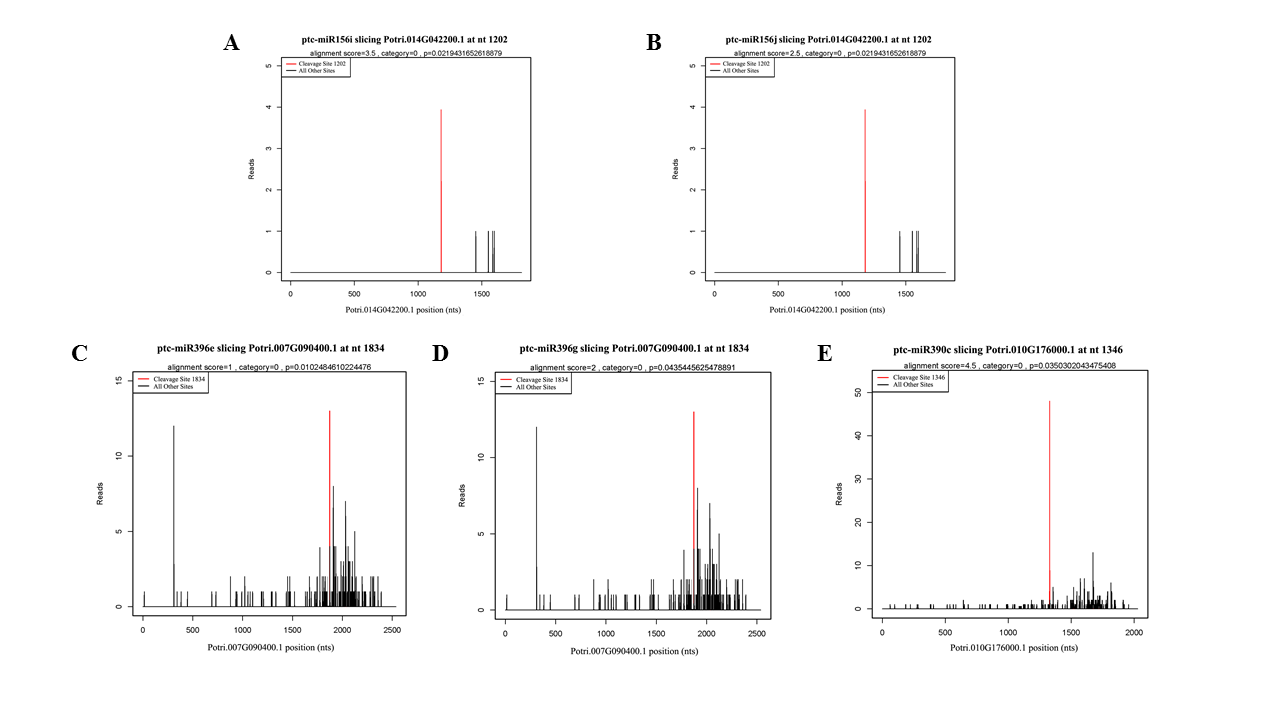


**Figure S4.** Target charts (t-charts) of microRNA targets confirmed by degradome sequencing during Populus simonii. The red line and black line represents the sliced-target site and mismatched RNA base pairs, respectively. A and B t-Charts are for *Potri.014G042200.1*. C and D t-Charts are for *Potri.007G090400.1*. E t-chart is for *Potri.010G176000.1*.


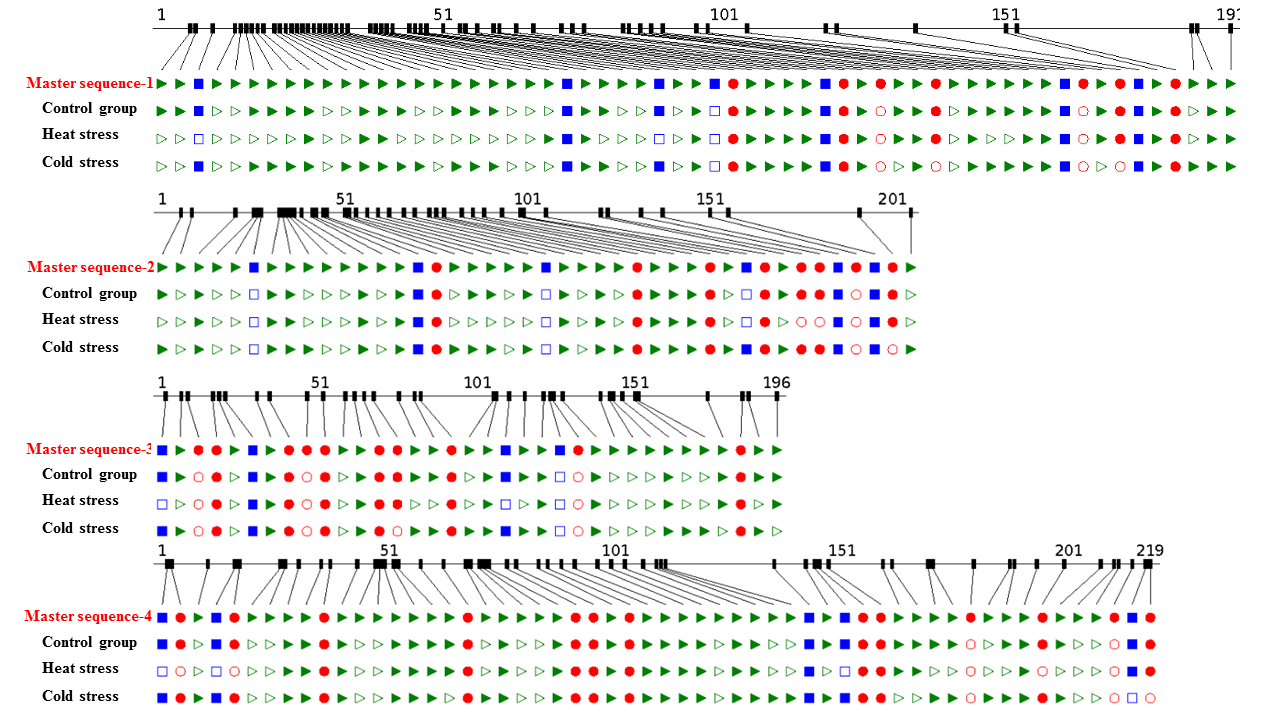


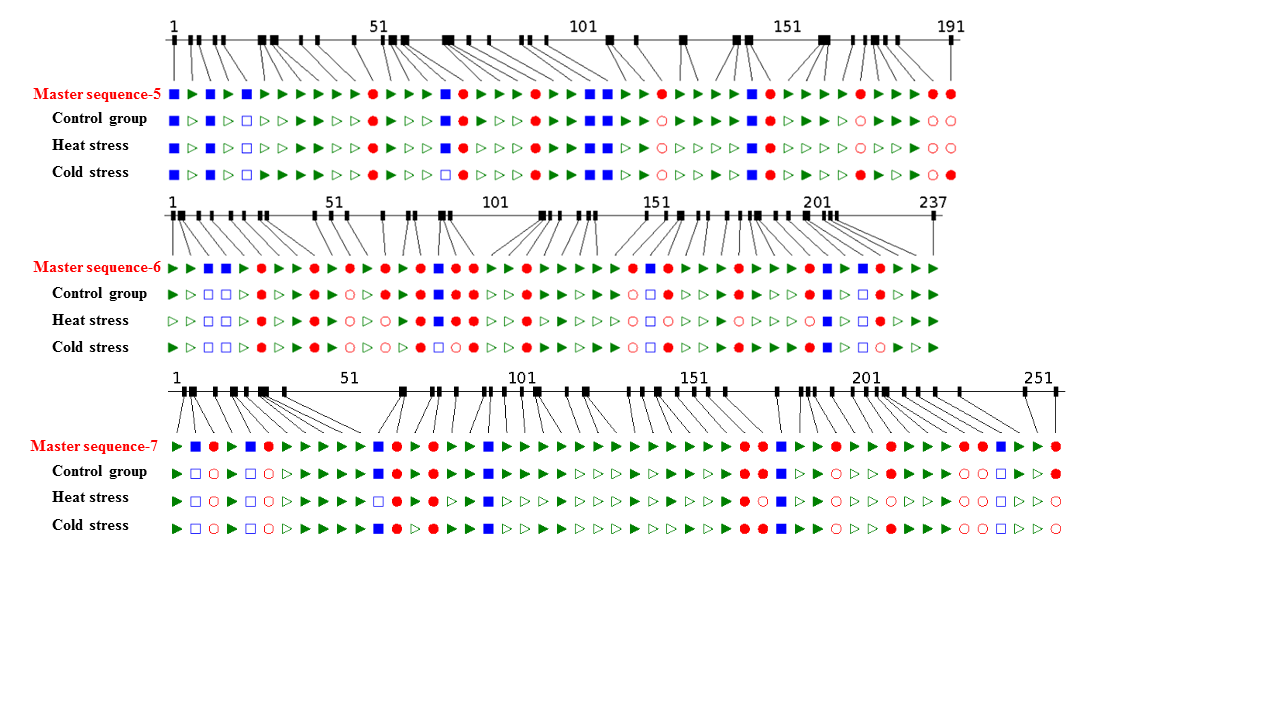


**Figure S5.** Abiotic stress-responsive DNA methylation patterns of candidate regions. ‘
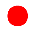
’ stands for ^m^CG; ‘
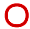
’ stands for CG; ‘
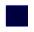
’ stands for ^m^CHG; ‘
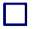
’ stands for CHG; ‘
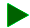
’ stands for ^m^CHH; ‘
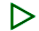
’ stands for CHH.

**Table S1. Information on realtime-PCR primer sequences of target genes**

| **Alias** | **Gene model** | **Sequence** | **Efficiency (%)** | |
| --- | --- | --- | --- | --- |
| SPL2 | Potri.001G055900 | F: ATCAGAGTATTGGAGGAC  R: GAGTTAATGGGAAGAAGA | | 98.1~103.9 |
| SPL9 | Potri.016G048500 | F: CTTCAACCCAGCACATCC  R: GAGCCAAAGAGTAATCATCC | | 97.9~102.1 |
| SPL2 | Potri.018G149900 | F: TTTCCAGGTTTGCTTTCA  R: ACTTGTTGCATCGTGCTT | | 99.7~108.1 |
|  | Potri.001G058600 | F: ATGCCATTTCTGGTTCTA  R: CATCCTGCGTTGACTTCT | | 98.5~101.7 |
| CAT5 | Potri.001G076100 | F: AGGCAGAATCAACAGAAG  R: CGTTACCGGACATTACAT | | 97.3~101.9 |
| ATVSR3 | Potri.001G294400 | F: AAGAGCTATCATGGCACA  R: TGGAACAAGGCAAGAGTA | | 97.5~100.7 |
| SPL5 | Potri.001G398200 | F: TCAGATAAACATCCCACC  R: AATAAGATTCCACCAAGC | | 97.9~102.1 |
|  | Potri.002G105400 | F: CGTTTGCGGTGTTTCTGA  R: GACCCAATGCGAGTGAGC | | 97.9~106.4 |
| SPL11 | Potri.002G142400 | F: GAGGGAAGAATAAAGAAGAG  R: TCAGGCTAGAAAGAAATACA | | 98.2~105.2 |
|  | Potri.002G169800 | F: AAGATGGGTCTCAGGAAT  R: CAGAAAGGGAAGTAGTTATT | | 95.9~104.9 |
|  | Potri.003G094000 | F: GCTGGAATAGCACTTGGA  R: CCGACTGGCATAAACCCT | | 98.8~105.6 |
| CAT5 | Potri.003G154600 | F: ATGGGATCTTTGGGTGCTG  R: CCGTTGATTGTGCCTTGC | | 98.9~105.4 |
|  | Potri.003G169400 | F: ACATGCCATTTCTAGTTC  R: CTTGGGTATCACATTCTAT | | 97.2~103.1 |
| SPL3 | Potri.004G046700 | F: AGGAGGTATGCTCACAGA  R: GAAGGGTAGGCAAAGTAG | | 99.1~101.9 |
|  | Potri.005G203100 | F：CTGCTGGATGTCTTTCTC  R：GTTCGGTTCCAAATCAATA | | 99.7~108.1 |
|  | Potri.007G137300 | F: CTGTTGATGCGTTGTAGT  R: CTGGAATAGTCACCTGCT | | 98.5~101.7 |
| SPL4 | Potri.007G138800 | F: CTCAAGCCGCAAAGTAAA  R: CTAGCAACCCTGACACCA | | 97.3~101.9 |
| TRFL9 | Potri.008G057300 | F：ACACGGACAGGAAACAGG  R：TTACAAATGCCAGCCTCA | | 99.7~108.1 |
|  | Potri.008G097900 | F: TATCGGTGCAGTAGACAA  R: AAGGGAGAAGAGCATTTA | | 97.9~106.4 |
|  | Potri.010G154300 | F: GTTTGATGTTGCTGGTTG  R: AATGAACAGAAGCGGACA | | 98.8~105.6 |
| SPL3 | Potri.011G055900 | F：TGGAGGAAGGCTCACTAC  R：TTTCTCGTCACCCAAATA | | 97.9~102.1 |
| SPL5 | Potri.011G116800 | F: CGTAGGCGTTTGGCAGGAC  R:TTCGCAGTTAAGCAAGAAGCAG | | 99.7~108.1 |
|  | Potri.012G017100 | F: TCGGCATCCAGCCAGAGT  R: CGCCACAAGGGTCACAAG | | 98.5~101.7 |
|  | Potri.012G100700 | F：CATAGGTCCAATAGTACATG  R：CTAGTTAGTGAAGATAATCCC | | 97.3~101.9 |
| AAT1 | Potri.012G131300 | F: GTTATGAATTTCCTACAC  R: ACATATCTAAATCCACCT | | 97.5~100.7 |
| CAT1 | Potri.013G030900 | F: CACGCCTCCTATGACACT  R: ATATCCCATCCACCACCC | | 98.2~105.2 |
| EDA9 | Potri.014G022800 | F：GTTAAAGAGGATCAGGGAA  R：CAAGAGGCAGTATGACAAT | | 95.9~104.9 |
|  | Potri.014G042200 | F: TATGTTGGGTATTTGCTC  R: GTTTCAAGGCATTCAGTAA | | 98.8~105.6 |
| SPL10 | Potri.014G057800 | F: TGTGACTTTGGACCTCAT  R: ATGCTCCTCTATAAACCTG | | 98.9~105.4 |
|  | Potri.015G060400 | F：ACGGTCAATCACCATCAT  R：AAGCGTTGAGAATAGAAGAG | | 97.9~102.1 |
|  | Potri.015G098900 | F: CAGTCTGGTGGTTCCTCT  R: CATGCCTAACTTGTTCATAG | | 99.7~108.1 |
|  | Potri.015G115300 | F: AACAAAGCATTAGCAAAAC  R: CATGATCCACGTAGAAGG | | 98.5~101.7 |
|  | Potri.017G015000 | F：CTTCAGCACCTCACCTCT  R：AACCTTTTATTGGCACAT | | 97.3~101.9 |
|  | Potri.017G015200 | F: ACCCCTAGTTCCTGATAT  R: TGGTATTTGTGCTTCTTT | | 97.5~100.7 |
|  | Potri.017G015300 | F: TCTCCCTTGTATCTTTTA  R: CAGTCTCAGCAACTTTAA | | 98.2~105.2 |
|  | Potri.017G015400 | F：TTTCAATGGCAAGTGGAT  R：TTGGGTGGGATTAGAGTT | | 95.9~104.9 |
| NLM5 | Potri.017G083300 | F: GGACCAGTGACAGGAACG  R: AAAGCAACACGGAAGATA | | 98.8~105.6 |
|  | Potri.019G069000 | F: ATGGCAACAGTAATGATAG  R: AAGAAAGAGGAGGTAGAT | | 98.9~105.4 |
| NRAMP6 | Potri.001G044900 | F：CTCAGAGGAGGAGTAATG  R：TCACTTAGTTCTACGAAAG | | 99.7~108.1 |
| sks5 | Potri.004G180500 | F: TGAATATCCAATTCCCAAGA  R: CCTGAAAATAGTAATGCAAACA | | 98.5~101.7 |
| SPL12 | Potri.014G114300 | F: TCAGGTGGGAATTGTTAG  R: ATGAGGAAATGAAGGAGA | | 97.3~101.9 |
|  | Potri.015G007400 | F：GGGCACCTTGATGTATGG  R：AGACCTGAAAGCGAGCAG | | 99.7~108.1 |
|  | Potri.019G057700 | F: TGCTGGAGATCACGAAGC  R: CATAGCGAGTGGCAAACC | | 97.9~106.4 |
|  | Potri.T134700 | F: TGCTGGAGATCACGAAGC  R: CATAGCGAGTGGCAAACC | | 99.7~108.1 |
|  | Potri.006G051700 | F：CTTAGCACCGTTTCCTTC  R：CTCTTCCGTCCATCCACC | | 98.8~105.6 |
| RLP21 | Potri.006G061300 | F: TATGCCATCCTGGTTAGA  R: TTGTCCCATTTGGTGTTA | | 98.2~105.2 |
|  | Potri.008G101700 | F: AGGAGCCTTATTTACCAT  R: CAACGAAACTGATGTCTG | | 95.9~104.9 |
| AIR3 | Potri.009G144500 | F：ATAGAGTAAGACGACCACC  R：CAATGACTTCTAGGCAAC | | 98.2~105.2 |
| ATPT2 | Potri.010G071600 | F: CCCAAAGAAGACTGACGC  R: CAAACACCGAGTGGTTGC | | 95.9~104.9 |
| ATPT2 | Potri.010G072000 | F: TATTGTGCTCGGTGTTAT  R: TAGCGACTACCTCCTTAT | | 98.8~105.6 |
|  | Potri.010G149600 | F：GTTACACTACAAGCCAGGAA  R：AGATGAGTTGGACGGAAA | | 98.9~105.4 |
| cICDH | Potri.010G176000 | F: CTATTTCCTCATCCGTTAT  R: CTGTCTTTCTTCGCTTTT | | 97.9~106.4 |
|  | Potri.011G073100 | F: CAGGAGGATTGCTGATAT  R: TAAGGCGATGAGTGAAGT | | 98.8~105.6 |
|  | Potri.011G073600 | F：TTTCTTGCAGCGACTTAA  R：GAATCCAATGATGGGTGT | | 99.7~108.1 |
|  | Potri.011G075400 | F: CTATCTCCGTGGTTGTGA  R: CGGTAAGTCCGTTGGTTT | | 98.5~101.7 |
| PSKR2 | Potri.011G116900 | F: CGTGGCTTGTATTGGATT  R: ATTGGGTTGGTTGACTGA | | 97.3~101.9 |
| ATPEPR1 | Potri.012G022800 | F：TGCTGAATATGCGTGAAA  R：AGAGGCATTTGGAAGGAA | | 97.9~102.1 |
| GSO1 | Potri.012G088900 | F：GGATACAGAAATCACAGGGAA  R：AAACTACAGGGTGGAGCA | | 99.7~108.1 |
|  | Potri.T008100 | F：ATGTGCTTAGTGGGAATA  R：TACCAGTTGTTGGGTAAT | | 98.5~101.7 |
|  | Potri.T008500 | F：CCTAAATTACACCCATCA  R：TTACTTGCAGCTTCACTC | | 97.3~101.9 |
|  | Potri.T008800 | F：AACTTCACTCATCGCCTTAC  R：AGCTTGTTGCCACTCAAA | | 97.5~100.7 |
|  | Potri.T009400 | F：AACAGTGCAGGACTCATT  R：ATAGACCATTATCCGTGA | | 98.2~105.2 |
|  | Potri.T009700 | F：CCATAACTAATGCCTGTA  R：TTGGGACTATGATAGAGG | | 95.9~104.9 |
|  | Potri.T009900 | F：ACCTGATTTGGTGGGATT  R：GCTTGATGGTGTTGTGGC | | 97.9~106.4 |
|  | Potri.T010100 | F：GCTCGCAACTATCTATACGG  R：TCCCAAGTGTCCAGGAAT | | 98.2~105.2 |
|  | Potri.T032300 | F：ACCGTACCCGAGTTCATT  R：TGGTCCAGGCATATCAGT | | 95.9~104.9 |
|  | Potri.T032600 | F：ATGCTTTGCCTTCTTTCC  R：TGCGGTGCTGTCAGTGGT | | 98.8~105.6 |
|  | Potri.T032800 | F：AATTTCTTGCAGCGACTT  R：GAATCCAATGATGGGTGT | | 98.9~105.4 |
| AFB2 | Potri.001G323100 | F：CCTGGCATTTCTTTGTTA  R：GCTTCTGATTCCGTTTCT | | 99.7~108.1 |
|  | Potri.001G413900 | F：GCAACAACGACCAGAAGA  R：TACAGTGGGCAGAAAGAA | | 98.5~101.7 |
| TIR1 | Potri.002G207800 | F：ACACTGAGCCTCAAGAGC  R：TTGGCAAACAGAACACTAA | | 97.3~101.9 |
|  | Potri.004G038500 | F：ATGAGGGTGATCAATCACTCAGAGG  R：CCTCTGAGTGATTGATCACCCTCAT | | 99.7~108.1 |
|  | Potri.005G147300 | F：CAGCTTCCACACTCAGAAACTCGAT  R：ATCGAGTTTCTGAGTGTGGAAGCTG | | 98.2~105.2 |
| ECT7 | Potri.008G100200 | F：AAAATTGCAGCTCTAACC  R：CCTCCTGGAATACATAAAA | | 95.9~104.9 |
|  | Potri.010G209300 | F：CTTGAGATGAAGACCCTG  R：GAGGTATAAAGACGCTTG | | 97.9~106.4 |
| TIR1 | Potri.011G042400 | F：TCCTGAAGAGCGAGTTTA  R：AATTAGGGTCCGTGAGAC | | 98.8~105.6 |
| TIR1 | Potri.014G134800 | F：CTGCTGGGTCAGAAGATG  R：TTCACTACAGGCAAGGGT | | 98.2~105.2 |
| AFB2 | Potri.017G061600 | F：TTTTAGCCTATTGGTGTTC  R：TTCGGAGTTCCATCATCT | | 95.9~104.9 |
| GRF3 | potri.006G115200 | F：CCTCCACTACCAAACTCA  R：AGCGTAACACGAAAGAAA | | 97.9~102.1 |
|  | Potri.001G017100 | F：AAAAGATACATTCGTTGC  R：GGAGGAAGTCACTAAAGC | | 99.7~108.1 |
| GRF8 | Potri.001G082700 | F：CCAAACTTATCCCATTCC  R：ACTTCCCATCAACTGCTA | | 98.5~101.7 |
| GRF2 | Potri.001G114000 | F：AGTAATGCCAGCACCAAC  R：AAGCCTGTAAATCATAACCA | | 97.3~101.9 |
|  | Potri.001G116300 | F：GATGCTAGTCTGCTCCAA  R：CTCCCTTCTGCTACTTTT | | 97.5~100.7 |
| GRF2 | Potri.001G132600 | F：TACTTTGAGATTGCGTGCTT  R：TAGGGTTTAGATTGATGCTTGT | | 99.7~108.1 |
| GRF5 | Potri.001G169100 | F：ACTTGTTCGTGGGTCATC  R：GGTGGTAGTAGGGAATGG | | 98.5~101.7 |
|  | Potri.001G215800 | F：TTCGACCACCTAAACTCA  R：ATGCTCAAGGGAAACTAA | | 97.3~101.9 |
| GRF5 | Potri.019G042300 | F：ATTTACAAAGCACGATCAAAGG  R：GTCTCACGCACCGCACAA | | 98.2~105.2 |
|  | Potri.018G127000 | F：TAAGCATGAGCCTTTGAT  R：CTCTGTCTGCCCAGTTAT | | 95.9~104.9 |
| GRF5 | Potri.018G065400 | F：TTGGATTATGTGGGTGTT  R：AGTAGGGTTCGTAGGTGA | | 98.8~105.6 |
|  | Potri.016G047700 | F：TTGGGTGATGGAACAATG  R：AACTTCTAATGCTGCCTA | | 98.9~105.4 |
| GRF7 | Potri.015G006200 | F：CAAGGACCAAACCTGAAA  R：ATCCGAAGAAGATCCAAGAA | | 98.2~105.2 |
|  | Potri.014G116600 | F：ACGACAGGAGGTTGATAC  R：ATGAGACAATGAGGGAAG | | 95.9~104.9 |
| PFC1 | Potri.014G077100 | F：CCTTGGATGATTTCGTTA  R：ATTGCTTTCAGCCTCTAT | | 99.7~108.1 |
| GRF9 | Potri.014G071800 | F：ATGCCTTCGTTGCTCTGT  R：AACAACCTGCTTTCTCAA | | 97.9~106.4 |
|  | Potri.014G024100 | F：CACCCAAAGATGGATGAT  R：ATGCCACAGATACAAGAA | | 95.9~104.9 |
|  | Potri.014G007200 | F：CCTGTAATCAAGCGAACT  R：AGCCGTGAGAAATAAGAC | | 99.7~108.1 |
|  | Potri.013G077500 | F：ACAAAGCCAAAGGTGGTC  R：GTCAAGGCAGGCGAGGTT | | 97.9~106.4 |
|  | Potri.012G058100 | F：CGATACCGTTCTAAGTCA  R：CAGCAGGTTGTTCTAATT | | 97.9~102.1 |
| GRF7 | Potri.012G022600 | F：AAATCAAGGCTAAATGTGC  R：CAAGTCTCCCTCGCTAAT | | 99.7~108.1 |
|  | Potri.011G045500 | F：CCCATGTATCTGAATGCT  R：CCTCGGCTGTAATAGTTT | | 98.5~101.7 |
|  | Potri.009G038300 | F：AATCGTGCTCTGAGTTGA  R：ACTGGTAGCGAGTGTTGT | | 97.3~101.9 |
| ACX2 | Potri.007G090400 | F：AAAGGGCAGTGAAGGATA  R：GATGAAACGAGCAACAATAA | | 97.5~100.7 |
| GRF1 | Potri.007G007100 | F：AGAATGTAAAGACTTGGCAGAT  R：GCGTGAATGATGGAGGTT | | 98.2~105.2 |
| GRF5 | Potri.006G143200 | F：AGATGTACTTGGTGGGAGG  R：TTCATATCAATGGCTTGG | | 95.9~104.9 |
| WSIP2 | Potri.006G066800 | F：GGACCTTTATTGTCTATGC  R：ATCTTCTGCTCGGAACTA | | 98.8~105.6 |
|  | Potri.003G116100 | F：TATCAGGGTGTAGTTGGA  R：CTAGATTTGGCTTTCTCA | | 99.7~108.1 |
| GRF2 | Potri.003G100800 | F：CCCAAATGGTGAGACAGG  R：CCCAAATGGTGAGACAGG | | 98.5~101.7 |
| GRF5 | Potri.003G065000 | F：AGTGGGAACCGAAGGATG  R：TACTGCCGTGGAGCGAAG | | 97.3~101.9 |
|  | Potri.002G197400 | F：GTGATGAATGTGGCTCTG  R：AGTGGCTAACTAGGAAGG | | 99.7~108.1 |
| GRF1 | Potri.002G115100 | F：TGTTGCTTGGCCTGGACT  R：TCTGCTCTTGGGCTGCTT | | 97.9~106.4 |

**Table S2. Information on miRNA realtime-PCR primer sequences**

| Alias | Gene model | Sequence | Efficiency (%) |
| --- | --- | --- | --- |
|  | ptc-MIR156i | TTGACAGAAGATAGAGAGCACA | 99.5~105.7 |
|  | ptc-MIR156j | TTGACAGAAGATAGAGAGCACG | 98.9~102.9 |
|  | ptc-MIR167h | TGAAGCTGCCAACATGATCTG | 99.7~104.1 |
|  | ptc-MIR390c | AAGCTCAGGAGGGATAGCGCC | 98.5~102.7 |
|  | ptc-MIR393a | TCCAAAGGGATCGCATTGATC | 98.7~103.1 |
|  | ptc-MIR396e | TTCCACAGCTTTCTTGAACTT | 98.9~104.1 |
|  | ptc-MIR396g | TTCCACGGCTTTCTTGAACTT | 97.8~103.2 |
|  | 5.8S rRNA | GTCTGCCTGGGTGTCACGCAA |  |
|  | Reverse primer | GTCGTATTAATTCTGTGCTCGC |  |
|  | Poly(T) adapter | GCGAGCACAGAATTAATACGACTCACTATAGG(T)12VN* |  |

*V = A, G, C; N = A, T, G, C

**Table S3. Information on PCR primer sequences of candidate differentially methylated sequences**

| **ID** | **Gene model** | **Sequence** | | **Efficiency (%)** |
| --- | --- | --- | --- | --- |
| **1**^a^ | ptc-MIR156i | F: GTGAACGCAATGAGAACG | 98.2~105.2 | |
|  |  | R: GGTAATATGTGGAAAGGAAAAT |  | |
| **2** | ptc-MIR156j | F: GCTTCTTTTCTTTTGGTC | 95.9~104.9 | |
|  |  | R: CTTTACAATGAACGGGAC |  | |
| **3** | ptc-MIR167h | F: TAAGGAAGGACCAATGTG | 98.8~105.6 | |
|  |  | R: TCTTGTTATTCCCTTGTG |  | |
| **4** | ptc-MIR390c | F: TTTTGGCAGGAAGGTATT | 98.9~105.4 | |
|  |  | R: TCGGTCGTTGGTAGAAAT |  | |
| **5** | ptc-MIR393a | F: ATCGTAAACTTTCCCTCC | 97.2~103.1 | |
|  |  | R: ACTCGGTATAATTTGAACAACC |  | |
| **6** | ptc-MIR396e | F: CCTCCTTCTTCTACTTTC | 99.1~101.9 | |
|  |  | R: ATCTCACCAGGTACTCTT |  | |
| **7** | ptc-MIR396g | F: TTCCTTTAGACCATTCTT | 99.7~108.1 | |
|  |  | R: CTTTTGGTTCTTTTATCC |  | |

^a^ The number represents candidate differentially methylated sequence (DMSs), seen in Excel S4
